# Reforming mental health in Lebanon amid refugee crises

**DOI:** 10.2471/BLT.16.030816

**Published:** 2016-08-01

## Abstract

The massive influx of Syrian refugees is galvanizing efforts to reform the mental health system in Lebanon. Rabih El Chammay talks to Fiona Fleck.

Q: How did the provision of mental health care start in your country?

A: It started in 1890 with the construction of an open psychiatric institution in a beautiful green area on a hill overlooking Beirut, called Asfourieh Hospital. With more than 46 buildings, it was the largest hospital of its kind in the region but was destroyed during the war in 1978. After that, two large psychiatric hospitals took the lead and still provide most mental health services in our country. However, in the absence of community-based services, many people with very few symptoms were staying in these hospitals for long periods of time. In recent years, university hospitals started opening psychiatric wards where patients could be admitted for short periods of time, so that they can be reintegrated into their families and communities again.

Q: How are you reforming the mental health system?

A: Historically, mental health services were dominated by the private sector providing psychiatric services, psychotherapy or hospitalization. To provide services for those who cannot afford the private sector, the health ministry launched the first national programme to reform the mental health system in 2014. Our vision is that everyone living in Lebanon should have the opportunity to enjoy the highest level of mental health and well-being. We are doing this in collaboration with WHO, UNICEF, the International Medical Corps and other partners. A major pillar of the reform is the integration of mental health care into primary health care. We are training doctors, nurses and social workers to identify, assess, manage and refer patients to mental health specialists based on WHO’s Mental Health Gap Action Programme (mhGAP).

“Everyone living in Lebanon should have the opportunity to enjoy the highest level of mental health and well-being.”

Q: What is new about the strategy?

A: The strategy was developed in line with the WHO *Mental health action plan 2013–2020*. It is evidence-based and takes a human rights approach. Two important new components are that we are addressing the needs of vulnerable groups (displaced populations, people in prison, the families of persons missing from the war, the survivors of torture and foreign domestic workers) and using a new approach to interpersonal psychotherapy. We aim to train all psychotherapists working on the Syrian crisis response in this approach. We are also piloting two new mental health interventions: one is a programme people can use by themselves online to help them cope with depression and the other consists of multi-disciplinary mental health teams to provide mental health care and psychosocial services in the community. Care in the community is important as it helps to prevent unnecessary hospitalization. The medicines we prescribe are being brought in line with WHO recommendations. We are also revising and developing much-needed laws and regulations for mental health services and for different mental health professionals.

Q: How is the Syrian refugee crisis affecting Lebanon’s public health efforts? To what extent is your country able to provide mental health care for the recent influx of refugees?

A: More than 1 million Syrian refugees are registered in Lebanon and there are another 500 000 or so who are not registered. Lebanon has a population of about 4 million Lebanese and 400 000 Palestinian refugees. So, on the one hand, this increase is a huge challenge for our health system, and on the other hand the massive influx of refugees has galvanized our efforts to respond. Now a great deal of energy is being put into developing our mental health system as part of the humanitarian response. In 2013, a UNHCR report concluded that there was a lack of coordination between agencies providing services to the refugees and that the inability to meet basic needs – such as food, water, shelter and health care – was the main source of tension between the refugees and their host communities. Our ministry set up a task force with UNICEF and WHO in 2013 to respond to these problems. Last year the ministry launched the new mental health care strategy that aims to provide care in the community for everyone living in Lebanon. That way, we avoid creating a parallel system for refugees that might collapse when humanitarian funding decreases. 

Q: What kind of mental health services do the Palestinian refugees in Lebanon receive?

A: Palestinian refugees have been living in Lebanon since 1948. Currently there are about 400 000 of them, most of whom live in camps in very difficult conditions. UNRWA is the agency responsible for basic services including education and health and for referrals to psychiatric hospitals. Now our health ministry is working with UNRWA to provide technical support to integrate mental health care into primary care and to make mental health services more widely available and accessible to Palestinians in need.

Q: How are you funding the new approach to mental health care?

A: Most of the funds are one-off payments for specific projects and therefore our funding sources are not sustainable. As the Syrian refugee crisis continues, there is potential for increasing external donor funding. The ministry’s mental health care budget is still geared towards hospitalization and medication. A key goal of the new strategy is to revise the budgetary allocations for mental health. This depends heavily on the political situation. Currently our country has no president and the parliament is not meeting. We hope this situation will change soon. The funds we receive are mainly channelled through our main partners, WHO, UNICEF and the International Medical Corps, with bilateral partners contributing as well.

Q: As a psychiatrist, which specialized therapies have you studied? Many of the specialized therapies have been tested and practised in Europe and North America. How relevant are they to people in the eastern Mediterranean?

A: I studied and practice several specialized approaches, including cognitive behavioural therapy and narrative exposure therapy. Such approaches are indeed applicable to people in Lebanon, but often they need to be adapted culturally. For example, we are adapting an e-health intervention for people with depression who can access the online mental health platform that we are currently preparing to launch. On this platform, there is the story of someone living with depression. This story is being carefully adapted to the local context in terms of customs and language. We will be consulting with Lebanese, Syrian and Palestinian native Arabic speakers – as each of these groups speaks a different dialect – to ensure the quality of the translation and content. The story was also peer-reviewed by mental health experts. Once ready, this e-health intervention will be piloted and we will conduct a randomized controlled trial to see whether the intervention is effective. This will take time, but it’s the only way to move forward.

Q: The national mental health programme document starts with the word “stigma”. How does stigma manifest itself in Lebanon and how do you deal with it?

A: Stigma and people’s lack of knowledge about mental disorders are two major barriers that prevent people from seeking help from professionals. For example, I work with a small nongovernmental organization in southern Lebanon, about 50 km south of Beirut, that attends to the mental health needs of Palestinian refugees and Lebanese host communities, people who are of low socioeconomic status and cannot afford to see a doctor in the private system. The stigma surrounding mental health is so great that some families prefer to take a day off, pay the transportation and the consultation fees so that they can come to see me in Beirut – all of which they can barely afford – rather than to see me at the clinic in the village. They are afraid that if people find out that their daughter or son is seeing a psychiatrist that he or she will have less chance of getting married. Stigma is mainly due to misconceptions. Many people mistakenly believe, for example, that people with mental health disorders have a weak personality, that they are not serious, that they will pass on a genetic defect to future generations, that people with mental disorders are always unreliable and violent, especially those with severe mental disorders. None of which is true. 

Q: How are you seeking to correct these misperceptions?

A: We want to develop public awareness of mental health disorders, and to advocate for the rights of people so that these misconceptions are addressed. We are preparing to launch a national campaign from 10 September to 10 October 2016 to raise awareness around depression and to encourage people to seek help, which is now available to them. In addition, we have a very active civil society in Lebanon that has been organizing annual campaigns to raise awareness around stigma and mental health for years now.

“Medicalizing human suffering is not the solution. Withholding medical treatment, because other major determinants should be tackled, is not either.”

Q: Do you have any last reflections about your work?

A: A huge dilemma that I still struggle with is that we are often acting in the aftermath of events. The mental health of people is affected so much by social determinants of health. Conflict, poor provision of basic needs and lack of safety are all beyond our control. Of course some refugees need mental health care, some because of the crisis and some because they already had mental health disorders and needed services anyway. Ensuring access to these services is of utmost priority in humanitarian settings. Sometimes the lines are blurred. Sometimes we, mental health professionals, have to ask ourselves: “Does this mother need an antidepressant or more money to care for her children?” Medicalizing human suffering is not the solution. Withholding medical treatment, because other major determinants should be tackled, is not either. There are no clear answers to these questions and it’s our duty as mental health professionals to deal with the grey areas and to be alert to the ethical and moral dilemmas we face, especially in complex emergencies.

